# A map of the extent and year of detection of oil palm plantations in Indonesia, Malaysia and Thailand

**DOI:** 10.1038/s41597-021-00867-1

**Published:** 2021-03-30

**Authors:** Olga Danylo, Johannes Pirker, Guido Lemoine, Guido Ceccherini, Linda See, Ian McCallum, Hadi, Florian Kraxner, Frédéric Achard, Steffen Fritz

**Affiliations:** 1 International Institute for Applied Systems Analysis (IIASA), Laxenburg, Austria; 2 KU Leuven, Department of Earth and Environmental Sciences, Leuven, Belgium; 3 UNIQUE Forestry and Land Use, Freiburg, Germany; 4 European Commission Joint Research Centre (JRC), Ispra, Italy

**Keywords:** Sustainability, Environmental impact

## Abstract

In recent decades, global oil palm production has shown an abrupt increase, with almost 90% produced in Southeast Asia alone. To understand trends in oil palm plantation expansion and for landscape-level planning, accurate maps are needed. Although different oil palm maps have been produced using remote sensing in the past, here we use Sentinel 1 imagery to generate an oil palm plantation map for Indonesia, Malaysia and Thailand for the year 2017. In addition to location, the age of the oil palm plantation is critical for calculating yields. Here we have used a Landsat time series approach to determine the year in which the oil palm plantations are first detected, at which point they are 2 to 3 years of age. From this, the approximate age of the oil palm plantation in 2017 can be derived.

## Abstract (summary)

**Table:** 

Measurement(s)	2-D extent • age
Technology Type(s)	Classification
Factor Type(s)	input dataset
Sample Characteristic - Organism	Elaeis guineensis
Sample Characteristic - Environment	oil palm plantation
Sample Characteristic - Location	Southeast Asia

Machine-accessible metadata file describing the reported data: 10.6084/m9.figshare.13899713

## Background & Summary

Driven by food and industrial demand, the production of global oil palm has more than doubled over the last two decades. Between 1997 and 2018, oil palm plantations expanded from 10 to 21 Million hectares (Mha), while crude palm oil production increased from 100 to 300 million tons (Mt)^1^. This rise has deeply impacted natural forest ecosystems^2–5^ and biodiversity^6,7^, while contributing significantly to climate change by releasing carbon from converted forests and peatlands into the atmosphere^2,8,9^.

Oil palm is known to be the most efficient oil-producing plant globally. Oil palm yields, however, vary dynamically with plantation stand age and location. They increase during the youth phase of the first seven years, reach a plateau during the prime age of 7–15 years, and then slowly start to decline before palms are replaced at the age of 25–30 years^10^. Therefore, knowing the exact extent and age of plantations across a landscape is crucial for landscape-level planning to allow for both sustainable oil palm production and forest conservation. Based on the land sparing theory^11^, the improvement of oil yields has been put forward as a means of reconciling oil production and forest conservation^12,13^.

The combination of high-resolution satellite records and cloud-computing infrastructures to handle big data can now be deployed to map oil palm extent and age, independent of official statistics. The latter are usually provided annually at a rather coarse spatial scale (national or regional administrative units), they may be incomplete or not updated regularly, and the quality is uncertain. Hence, they do not provide detailed spatial extent or age. Several recent studies have applied remote sensing techniques to estimate the extent of oil palm plantations in Southeast Asia. Studies aimed at quantifying plantation extent on a national scale or larger have deployed visual, expert-based interpretation methods^2–5,14^ or semi-automatic approaches^15^, blended with extensive field information^16,17^. These approaches are, however, very labour intensive.

The availability of radar imagery has given rise to a new generation of remote sensing studies benefiting from penetration of clouds, improved resolution and high revisiting frequency^18–20^. Recent experimental studies have demonstrated the usability of the European Union’s free and open Copernicus Sentinel data to detect oil palm plantations^21–23^. Sentinel data have proven particularly useful in detecting smallholder and industrial plantations^22–24^ and plantation age^25^, which, in turn, is a good predictor of oil palm yields^10,25^.

Here we have mapped the extent and the age of *productive* oil palm plantations in Indonesia, Malaysia and Thailand, the three countries that in 2017 contributed to almost 90% of global oil palm production^1^, at a 30 m resolution using remote sensing. *Productive* oil palm plantations comprise both industrial and smallholder plantations that have an age of at least three years. We have excluded roads, mill infrastructure and other land uses occurring in oil palm-dominated landscapes. The methodology for determining the extent of plantations builds on Sentinel-1 microwave data whereas the Landsat archive has allowed us to build a time series reaching back to the 1980s to determine when the plantation was established. The methodology identifies oil palm plantations of all types independent of weather and daylight conditions or ground-sourced information amidst other tree crops in the landscape. We determined the accuracy of the resulting oil palm map against an independently collected and interpreted sample of very high-resolution satellite images.

## Methods

The main input data used in the detection of oil palm include: (1) the Copernicus Sentinel-1 microwave backscattering time series; (2) the Landsat multispectral time series; and (3) additional auxiliary data sets. All data acquisition and processing were performed using the Google Earth Engine (GEE)^26^.

### Oil palm plantation detection

Oil palm area detection requires preparation of Sentinel-1 data and the application of an unsupervised oil palm detection routine based on the Normalized Difference Vegetation Index (NDVI) and texture features. The combination of NDVI and texture is important to reliably distinguish oil palm from other woody vegetation. To this end, an annual mosaic of backscatter coefficients for 2017 was produced by processing all available Sentinel-1 data (1A and 1B) with single polarizations (VV and VH; V is vertical, H is horizontal). The main objectives in producing this annual mosaic were: (i) to build a baseline C-band backscattering coefficient mosaic of Southeast Asia; and (ii) to assess radar backscatter changes between different years in land cover change studies.

The value of the output pixels corresponds to the ‘mean’ value of the Sentinel-1 collection after: (1) outlier removal (eliminating the lowest 20% of values); and (2) normalization for incidence angle to remove some of the ascending/descending variation. In addition to VV and VH yearly means, we added the VH/VV difference and the summed average (SAVG) texture metrics from the Grey Level Co-occurrence Matrix around each pixel of every band (radius of three pixels). To calculate the SAVG, all input data were scaled to bytes.

**Fig. 1:**
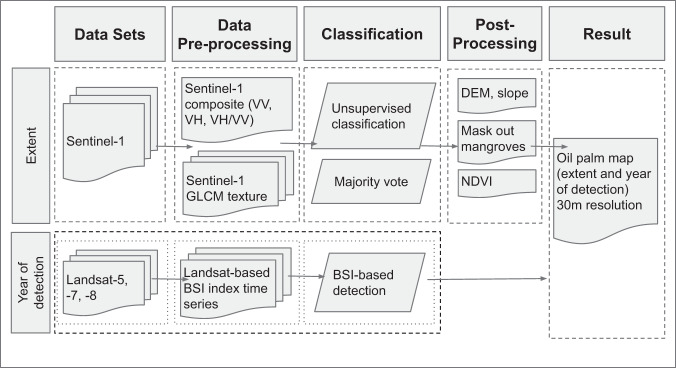
Workflow to produce the oil palm map (extent and year of detection).

We deployed a stratified unsupervised classification algorithm to detect oil palm without training data; a conceptual overview of the workflow is presented in Fig. 1. First, to account for regional differences, we divided Southeast Asia into 12 equally sized grids with a side length of five degrees and randomly allocated 50,000 training points. We used unsupervised classification with yearly means of VV, VH and the VV/VH-ratio derived from Sentinel-1 images as input features and the SAVG texture metrics from the Grey-Level Co-Occurrence Matrix of those features. For each grid, the detection algorithm returns the optimal number of clusters (between 10 and 16) to fit the sample; each cluster represents one land cover class (or what the algorithm recognizes as having a similar pattern). Subsequently, we identified those clusters that correspond to oil palm areas by comparing them with very high-resolution imagery. We then applied the oil palm classification algorithm from each grid to the entire study area, resulting in twelve, at times contradicting, oil palm maps. We reconciled these competing classifications by applying a majority filter, i.e., the final product shows oil palm where seven or more of the 12 classifications identify oil palm.

Post-processing was used to apply rule-based corrections to the intermediate oil palm map resulting from the unsupervised classification. These corrections were necessary in settlements, mangroves and sloping areas, as well as for other objects like roads running through the plantations. From a reflectance and backscattering viewpoint, settlements are very heterogeneous, such that the backscattered values in some parts of settlements are similar to those observed from oil palm fields. Setting a minimum NDVI value of 0.5 removed settlements from the area classified as oil palm. Similar confusion exists with mangroves. This was resolved by deploying a mangrove mask, derived from three regional and global mangrove maps^27–29^, and removing areas falsely classified as oil palm from within the mangrove area.

On Kalimantan, coverage by Sentinel satellites is in descending orbit only, i.e., imagery is always taken from the same perspective. This results in gaps, notably in hilly areas. To account for these gaps on south sloping areas in Kalimantan, we applied a post-processing majority filter within a 3 × 3 (30 × 30m) moving window to fill gaps in detected oil palm plantations. This process introduced a *de facto* minimum mapping unit of 900 m^2^ for the oil palm map. For a refinement of the final product, we removed pixels with NDVI less than 0.5, which at times have been erroneously introduced by the majority filtering in small non-palm areas such as roads running through plantations. We used digital elevation data from the Shuttle Radar Topography Mission (SRTM) at a resolution of one arc-second (approximately 30 m) to define terrain slope. This was required to correct the Sentinel 1 signal with respect to hillshades^30^.

### Oil palm plantation stand age detection

As an oil palm canopy develops over time, the area fraction of bare soil decreases, which can be detected with optical remote sensing through the bare soil index (BSI). To estimate plantation stand age, we moved backwards in time from oil palm plantations detected in 2017 until bare soil exceeded a certain value in young stands. For this we used surface reflectance values from the Landsat 5 and Landsat 7 image collections. We masked out clouds for every scene using the pixel quality attributes generated from the CFMASK algorithm^31,32^. We then generated a time series of BSI development starting from 1984. To smooth the time series and to remove noisy observations, we calculated the median BSI from a moving window of 12 months of the time series and omitted any 12-month time period in which we had less than three observations.

To determine a reference BSI for oil palm, we calculated a histogram of BSI values from established plantations in 2017 and used the 95^th^ percentile as a threshold value. The 95^th^ percentile coincides with the presence of very young plantations (two to three years of age with an open canopy and a high BSI), as confirmed by the visual interpretation of high-resolution imagery. For all pixels above the threshold in 2017, we analysed the BSI time series backwards in time where canopy closure is defined as the point when the BSI index drops below the cut-off value. This means that the resulting oil palm age map will register the first observation of oil palm plantations at an age of two to three years.

## Data Records

The data set is publicly accessible for download from the permanent DARE repository housed by the International Institute for Applied Systems Analysis (IIASA) (http://dare.iiasa.ac.at/85/)^33^. It consists of a 16-bit GeoTIFF at a resolution of 30 m with a single attribute value, i.e., the year in which the oil palm plantation was first detected. At this point, the plantation is 2 to 3 years of age. The data values range from 0 to 37 where 0 is the No Data value. Values 1 to 3 are not present and a value of 4 corresponds to the year 1984, the first year oil palm was detected, and each consecutive number represents the next year, i.e., 5 is 1985, while the maximum value of 37 corresponds to 2017. Table 1 provides a summary of the extent of oil palm plantations in 2017 by country, with the percentage that was planted before 2000 and the percentages planted between 2000 and 2009 and between 2010 and 2017, showing the acceleration in planting over time.

**Table 1:** Extent of oil palm plantations in the year 2000 and 2017 and the corresponding area increase during this period.

Region	Extent in 2017 (Mha)	Planted before 2000 (%)	Planted 2000–2009 (%)	Planted 2010–2017 (%)
Sumatra	6.37	18.97%	37.90%	43.13%
Kalimantan	2.92	7.01%	32.85%	60.14%
Peninsular Malaysia	2.41	17.31%	39.04%	43.66%
Insular Malaysia	1.72	22.34%	31.35%	46.30%
Thailand	1.06	10.44%	25.51%	64.05%

**Fig. 2:**
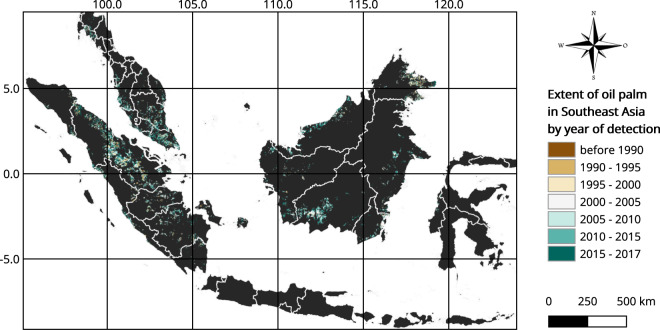
An overview of the extent and age of detection of oil palm plantations in Indonesia, Malaysia and Thailand.

Figure 2 shows the location of the remotely sensed oil palm plantations in Indonesia, Malaysia and Thailand in 2017, classified by year of planting.

**Fig. 3:**
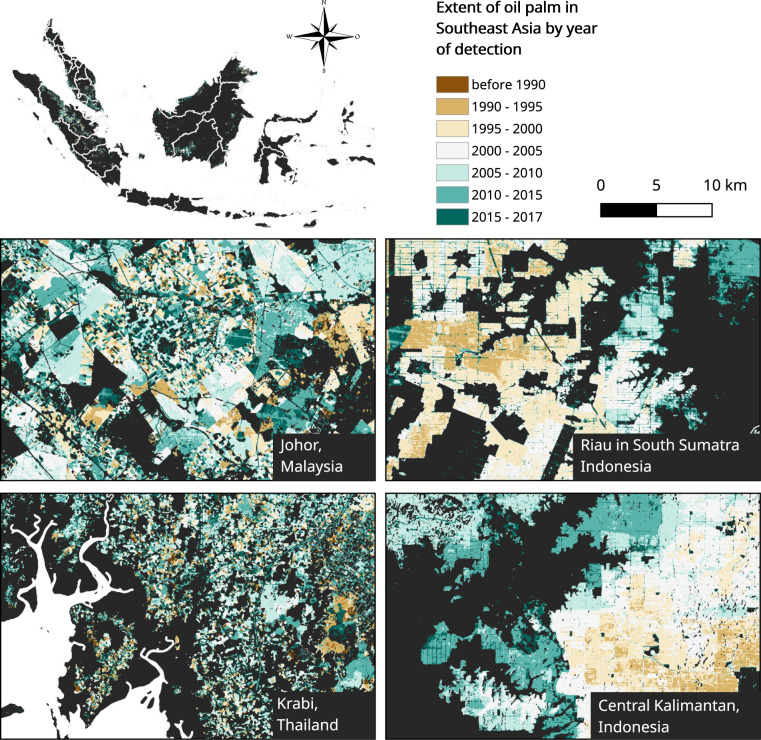
The extent and year of detection of oil palm plantations zoomed into four locations: Krabi, Thailand, Johor in Malaysia, Central Kalimantan and Riau in South Sumatra, Indonesia.

Figure 3 shows zoomed in locations of remotely sensed oil palm plantations in Indonesia, Malaysia and Thailand, classified by year of planting.

## Technical Validation

### Validation of the oil palm plantation map

We validated the oil palm plantation map using visual interpretation of very high-resolution satellite imagery with a random stratified sample. Agreement and disagreement with three other oil palm maps^3,19,34^ determined the three strata^35^: we placed 25% of the validation points in the stratum where all three maps agree on the presence of oil palm, 25% where all three maps agree on the absence of oil palm, and the remaining 50% of the points in the areas of disagreement among any one of the other three maps. This is a conservative approach to accuracy assessment as the focus is on areas that are difficult-to-map, which will most likely reduce the accuracy numbers compared to other approaches such as that adopted by Descals *et al*.^22,23^.

**Fig. 4:**
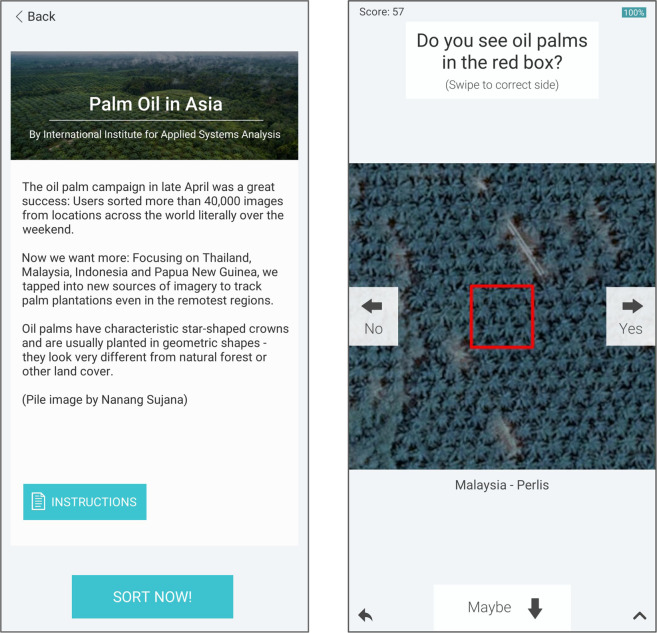
The Picture Pile application for collection of validation data. (**a**) The user instructions and (**b**) a very-high resolution image presented to a user, asking them to classify an image as oil palm by swiping to the right for yes, left for no and down for maybe.

For each randomly located sample, we downloaded an image from Bing Maps or Google Maps using an automated workaround implemented in R. After removing those samples where very-high resolution images were not available, 44 independent citizen scientists performed visual interpretation of the 8,609 sites with the available very-high resolution imagery. The online tool used for validation was Picture Pile (https://geo-wiki.org/games/picturepile/^36^), where volunteers evaluated whether oil palm was present or not at each sample location (Fig. 4).

**Fig. 5:**
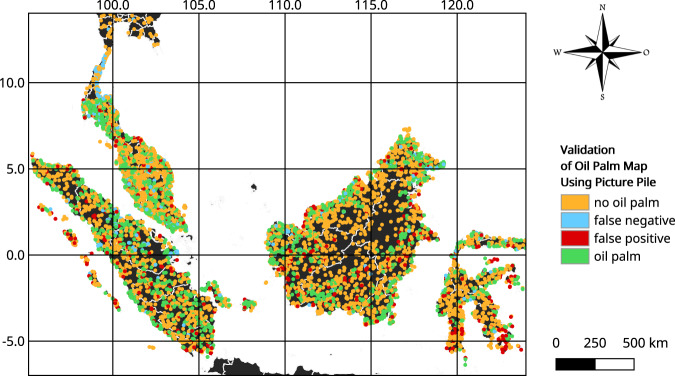
A map of the results of the validation procedure across South-East Asia. Colours of the points indicate agreement of remotely sensed and visually interpreted classifications. Green - Oil palm - hit; Orange - No oil palm - correct rejection; Blue - No oil palm - miss; Red - No oil palm - false detection.

Each image was assessed 5 to 8 times by different users, allowing for cross-user comparison of the same image. We only used those validations where there was 80% or more agreement among users. The results are mapped in Fig. 5, which were then used to generate the validation metrics presented in Table 2 based on the method outlined in^37^. Previous data collection campaigns with Picture Pile have shown high accuracies in identifying cropland^38^ and building damage assessment^36^. The validation data are available as a shapefile from the Github repository: https://github.com/odanylo/oilpalmseasia, which also contains the code. Although the majority of the imagery used in the validation was dated between 2015 and 2019 based on a sample of images selected, some of the imagery used in the validation was older. We acknowledge the limitation of this approach due to the temporal availability of very high-resolution satellite imagery, which may contribute to some omission errors.

**Table 2:** Map accuracy metrics assessed using independent validation samples.

Region	# samples	Accuracy [%]	95% CI [%]	Producer’s Accuracy [%] No OP/OP	User’s Accuracy [%] No OP/OP
Kalimantan	1,444	82.20	80.13–84.14	75.38/94.40	96.01/68.20
Sumatra	1,325	84.83	82.78–86.72	80.62/90.21	91.31/78.48
Sulawesi	1,206	77.28	74.81–79.62	71.41/86.32	88.93/66.24
Insular Malaysia	1,044	84.58	82.24–86.72	83.25/86.16	87.73/81.23
Peninsular Malaysia	1,500	87.07	85.26–88.72	86.60/87.45	85.09/88.75
Thailand	1,413	82.09	79.99–84.06	80.30/84.38	86.77/77.06
Summary	7,932	83.11	82.26–83.93	79.20/88.07	89.39/76.92

No OP = No oil palm; OP = Oil palm; CI = Confidence interval.

A further comparison was undertaken between the map produced here and the one from Descals *et al*.^23^. We randomly distributed 115,000 points across regions in common between both maps. Based on a binary classification of oil palm/non-oil palm, the overall agreement was 95.7%. Hence there is good general agreement between these two products although we additionally provide age of detection. We do, however, acknowledge that the age has not been validated in the same way as oil palm extent using visual interpretation, as this was not possible due to the lack of sufficient very high-resolution imagery to carry out a rigorous accuracy assessment. Instead we provide an example in Figure S1 in the Supplementary Information that provides time series of NDVI and BSI, indicating the age of detection in 2012. Very high-resolution satellite imagery is also provided to show forest before 2012 and oil palm in subsequent years after 2012. Similar manual verification was carried out at other locations to demonstrate that the method worked.

We acknowledge that there is some confusion between oil palm and coconut, which will lead to possible commission errors. These patterns are visible when we overlaid polygons of coconut plantations^39^ onto our map and the map of Descals *et al*.^23^. The confusion that is present in both maps can be viewed in more detail using GEE: https://olhadanylo.users.earthengine.app/view/danylovsdescals

## Usage Notes

Complementing national inventories with the map produced here could support the calculation of spatially-explicit estimates of greenhouse gas emissions and removals^40^, while also allowing for an independent check of the official statistics. The oil palm map in combination with spatial information about estate boundaries would allow specific actors and their adherence to environmental legislation and compliance with sustainability standards to be identified. The oil palm map could also be used in analyses related to determining the economic trade-offs in different types of land use.

Plantation stand age is an important predictor of oil yield as palm age influences the quality and quantity of the fresh fruit bunches^10^. Palm oil yield development follows an archetypical pattern determined by the plantation life cycle; it is characterised by a rapid growth, followed by a peak and a subsequent steady decline of productivity where productivity of 25–30 year old palms is estimated to be 60–90% of peak productivity and decreasing further after this stage. In that sense, renewing oil palm stands at an age of around 25 years is one potential contribution towards reducing oil yield gaps, notably in the oldest production areas such as parts of Malaysia.

Woittiez *et al*.^41^ highlight a number of causes of yield gaps (in decreasing order of importance for SE-Asia): the choice of the right variety (dura vs. tenera); inadequate water management, notably on peat soils; inadequate fertiliser doses, in particular potassium; diseases; and labour shortages. Furthermore, interlinkages exist between overaged plantations and these causes: high-yielding tenera varieties have been becoming more widely used over time, meaning that old plantations still contain higher fractions of dura. Many diseases tend to strike harder on old plantations and harvesting of older and hence higher palms requires more labour.

The remote sensing product presented here can be used to estimate productivity and assist high-level planning of the oil palm sector to adapt its strategies regarding plantation management, e.g., with respect to the need for renewal of aging plantations as highlighted by Wahid and Simeh^42^.

Finally, the generation of such a data set in near real-time could provide timely, independent, transparent and consistent monitoring of palm oil production across large geographical areas, bridging the gap between technology and land policy.

## Supplementary information

Supplementary Information

## Data Availability

The remotely sensed oil palm map is available to view in GEE at https://olhadanylo.users.earthengine.app/view/oilpalmseasia. The GEE code, documentation and validation data are available in Github at: https://github.com/odanylo/oilpalmseasia.
