# Author Correction: A map of the extent and year of detection of oil palm plantations in Indonesia, Malaysia and Thailand

**DOI:** 10.1038/s41597-021-00917-8

**Published:** 2021-05-05

**Authors:** Olga Danylo, Johannes Pirker, Guido Lemoine, Guido Ceccherini, Linda See, Ian McCallum, Hadi, Florian Kraxner, Frédéric Achard, Steffen Fritz

**Affiliations:** 1 International Institute for Applied Systems Analysis (IIASA), Laxenburg, Austria; 2 KU Leuven, Department of Earth and Environmental Sciences, Leuven, Belgium; 3 UNIQUE Forestry and Land Use, Freiburg, Germany; 4 European Commission Joint Research Centre (JRC), Ispra, Italy

**Keywords:** Sustainability, Environmental impact

Correction to: *Scientific Data* 10.1038/s41597-021-00867-1, published online 30 March 2021

The original version of this Data Descriptor incorrectly referred to the *RESTORE*+ *project* in the Acknowledgements section and mention of the *EU’s Framework 7 European Research Council project CrowdLand* and *EU Horizon 2020 project WeObserve* were omitted. This has now been corrected in the PDF and HTML versions of the Article.

