# Supplementary material for: A map of the extent and year of detection of oil palm plantations in Indonesia, Malaysia and Thailand
Source: Sci Data. 2021 Mar 30;8:96. doi: 10.1038/s41597-021-00867-1 (PMC8010082; doi:10.1038/s41597-021-00867-1)
Supplement: Supplementary file 1 — Supplementary Information [file 41597_2021_867_MOESM1_ESM.docx]

**Supplementary Information for A map of the extent and year of detection of oil palm plantations in Indonesia, Malaysia and Thailand**

### **Authors**

Olga Danylo^1^, Johannes Pirker^1,2^, Guido Lemoine^3^, Guido Ceccherini^3^, Linda See^1^, Ian McCallum^1^, Hadi^1^, Florian Kraxner^1^, Frédéric Achard^3^, Steffen Fritz^1^

**Affiliations**

1 Ecosystems Services and Management Program, International Institute for Applied Systems Analysis, Schlossplatz 1, A-2361, Laxenburg, Austria.

2 KU Leuven, Department of Earth and Environmental Sciences, Leuven, Belgium

3 European Commission, Joint Research Centre, I-21027 Ispra (VA), Italy

Corresponding author: Olga Danylo (danylo@iiasa.ac.at)

115.085884 -1.845399

| 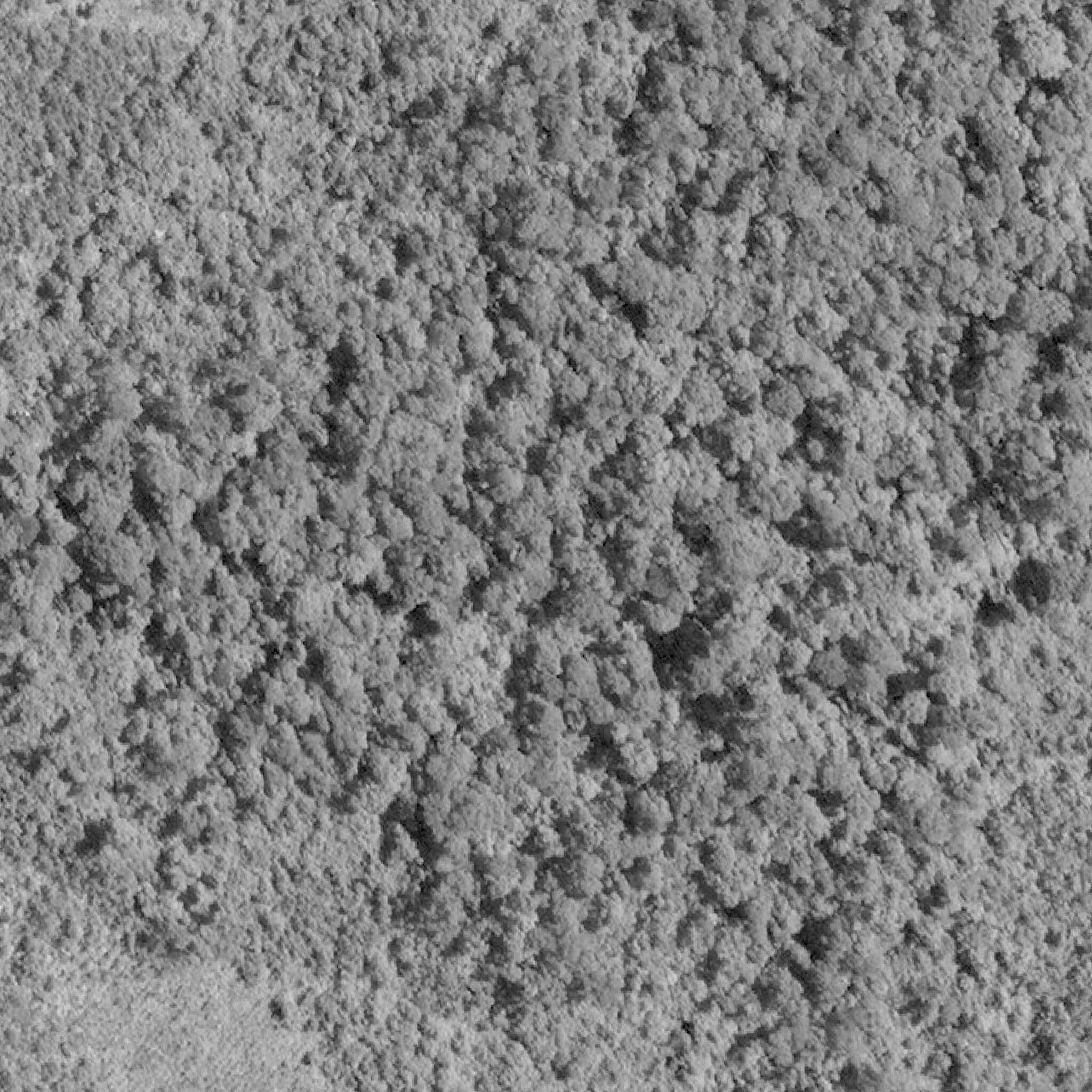  2008/06/22 | 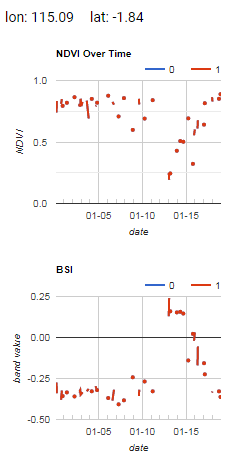 |
| --- | --- |
| 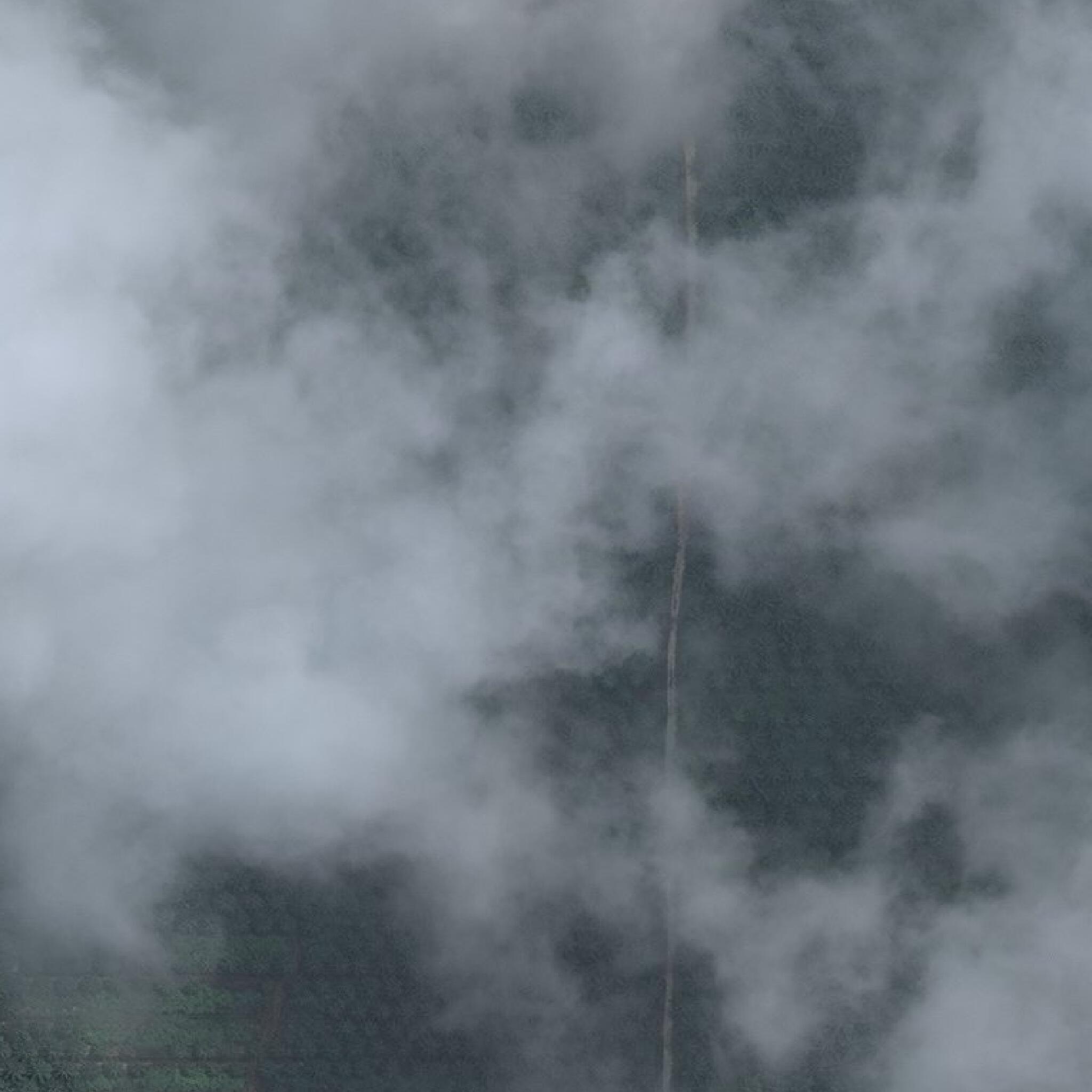  2016/07/05 |  |
| 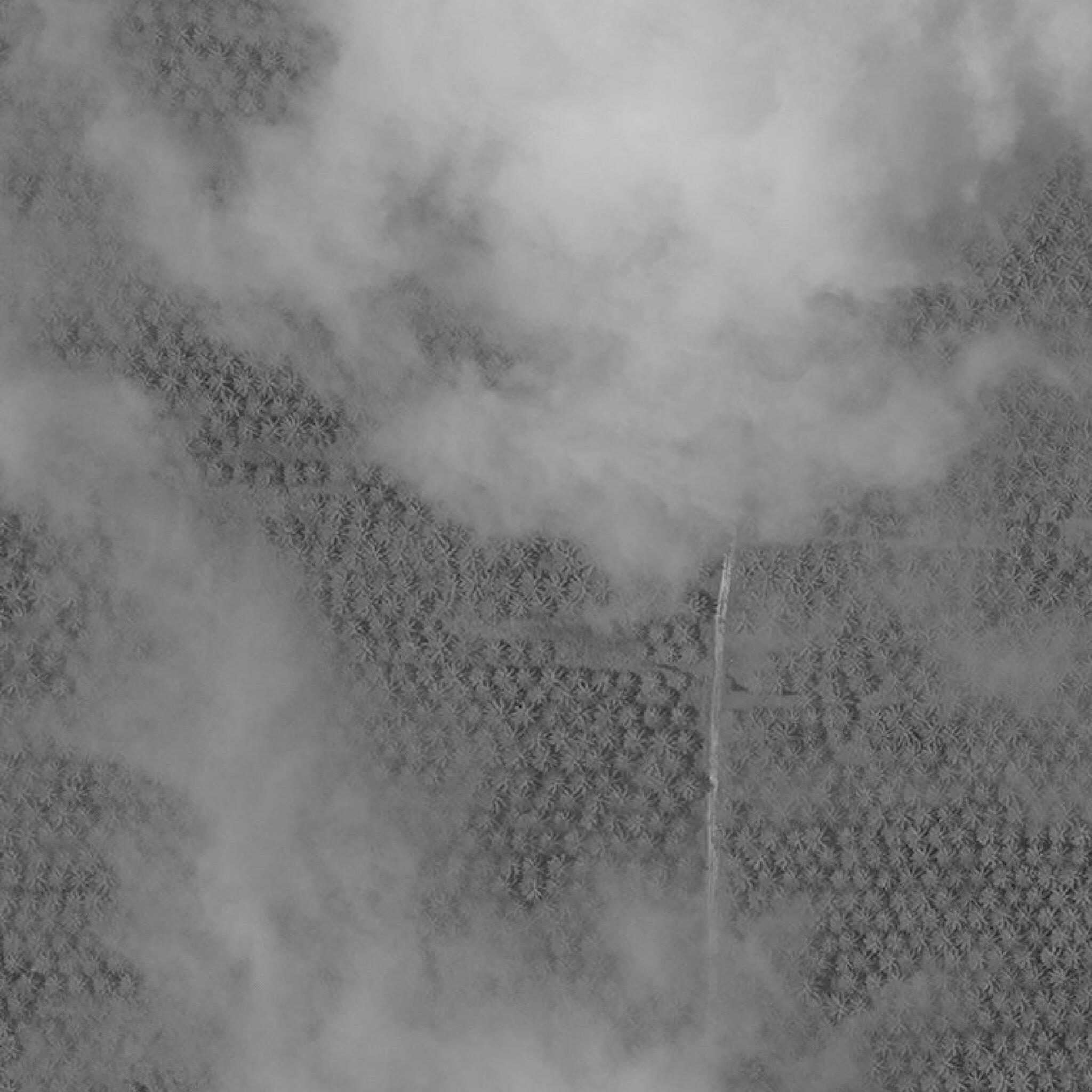  2017/10/13 | 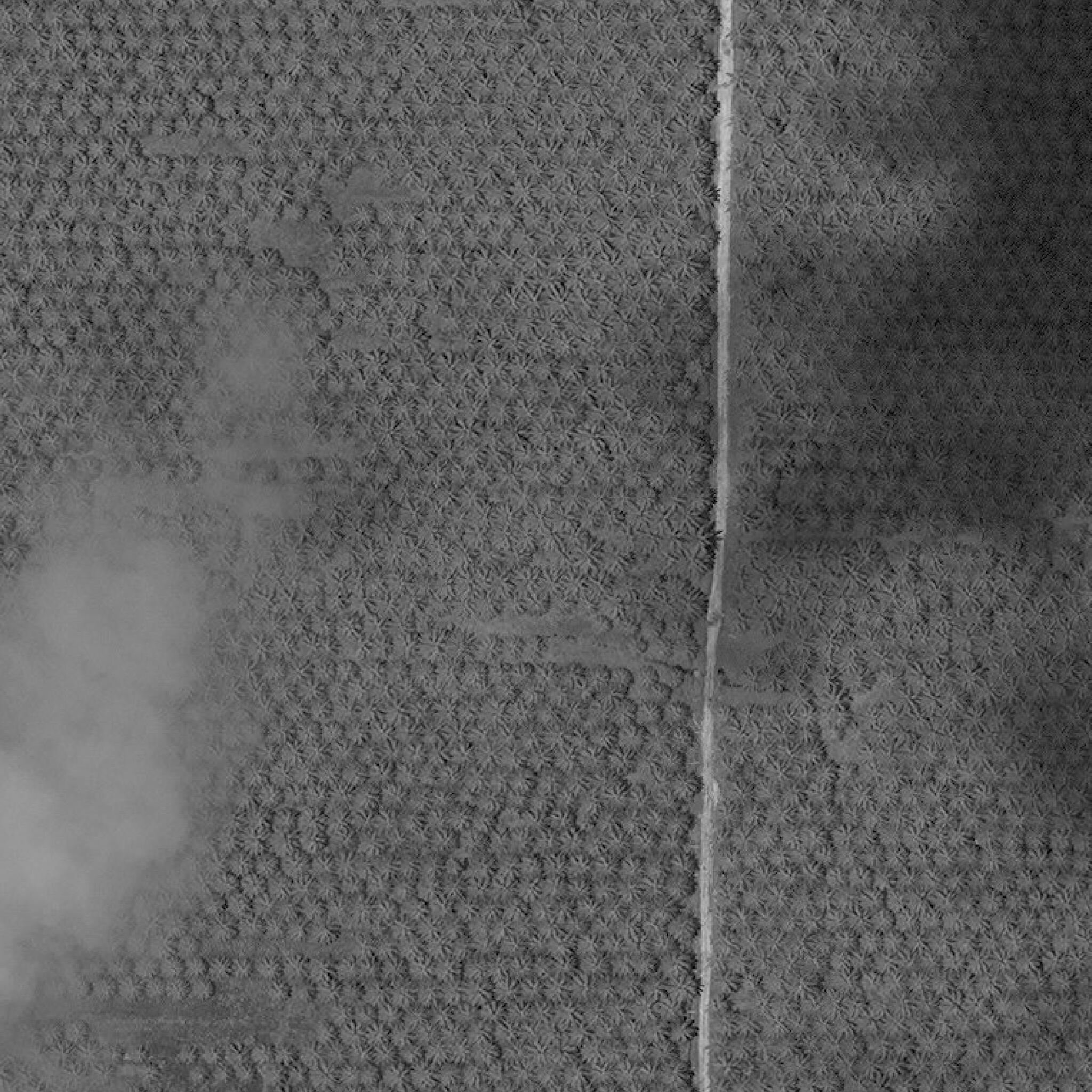  2018/03/11 |

Figure S1: Very high-resolution satellite imagery from Google Maps of a sample location, changing from forest in 2008 to oil palm in 2016, 2017 and 2018. Time series of the Normalized Difference Vegetation Index (NDVI) and the Bare Soil Index (BSI) indicate the year of detection as 2012.
